# Cancer and intercellular cooperation

**DOI:** 10.1098/rsos.170470

**Published:** 2017-10-04

**Authors:** Marta Bertolaso, Anna Maria Dieli

**Affiliations:** 1 Departmental Faculty of Engineering and FAST Institute for Philosophy of Scientific and Technological Practice, Università Campus Bio-Medico di Roma, Roma, Italy; 2 Department of Literature, Philosophy, and the Arts, University of Rome Tor Vergata, Roma, Italy; 3 Institute for the History and Philosophy of Science and Technology (IHPST), Paris 1 Panthéon-Sorbonne University, Paris, France

**Keywords:** cancer, natural selection, evolutionary transitions, multilevel selection, cooperation, inclusive fitness

## Abstract

The major transitions approach in evolutionary biology has shown that the intercellular cooperation that characterizes multicellular organisms would never have emerged without some kind of multilevel selection. Relying on this view, the Evolutionary Somatic view of cancer considers cancer as a breakdown of intercellular cooperation and as a loss of the balance between selection processes that take place at different levels of organization (particularly single cell and individual organism). This seems an elegant unifying framework for healthy organism, carcinogenesis, tumour proliferation, metastasis and other phenomena such as ageing. However, the gene-centric version of Darwinian evolution, which is often adopted in cancer research, runs into empirical problems: proto-tumoural and tumoural features in precancerous cells that would undergo ‘natural selection’ have proved hard to demonstrate; cells are radically context-dependent, and some stages of cancer are poorly related to genetic change. Recent perspectives propose that breakdown of intercellular cooperation could depend on ‘fields’ and other higher-level phenomena, and could be even mutations independent. Indeed, the field would be the context, allowing (or preventing) genetic mutations to undergo an intra-organism process analogous to natural selection. The complexities surrounding somatic evolution call for integration between multiple incomplete frameworks for interpreting intercellular cooperation and its pathologies.

## 1. Introduction

Most theories and models of cancer have been expanding, at some point, their explanatory accounts into an Evolutionary Somatic view of cancer, which conceives the disease as a result of progressive intra-organismal natural selection of the most malignant cells, coupled with the progressive accumulation of mutations especially in tumour suppressor genes (TSGs) and oncogenes (ONGs) [1,2]. Tumour progression would proceed according to a process that is similar to neo-Darwinian evolution, where each genetic change confers some selective advantage for cell growth and where genetic instability, a common feature in many cancers, would constitute an ‘enabling characteristic’ that facilitates the acquisition of additional mutations due to the damage that a cell could have previously undergone to its DNA repair system. A mutation of an ONG or TSG would be followed by the expansion of a benign tumour; additional mutations would lead to the primary tumour, then to its expansion, through the loss of genomic integrity of the cells, and ultimately to tumour transformation, from benign to malignant.

Hanahan & Weinberg [3,4] moved along these premises when they suggested in their classic article, ‘The Hallmarks of Cancer’, that cancer can be described by a small number of functional ingredients, despite the complexity of the pathology.^1^ A cell at the end of a clonal cumulative selective retention of mutations can acquire all six ‘hallmarks of cancer’, finding itself more competitive than the others that have not (yet) acquired them all.

Empirical studies of genetic mutations and of their possible effects on the cell have explored many possibilities. For one, the mutations that accumulate stochastically within the expanding populations of clones occur not only at the level of ONGs and TSGs, but may also involve other genes. Furthermore, authors who are most aware of evolutionary theory reject the view of step-wise accumulation of mutations as simplistic and inaccurate [5]. A conventional Darwinian framework is more properly formulated as ‘selection acting on heritable diversification’ in natural populations. Nonetheless, a gene-centric version of Darwinian evolution is the most frequently evoked in general discourses and empirical studies of cancer. Now, thanks to significant theoretical advances, the evolutionary possibilities of a somatic cell are understood within a conceptual framework based on the system-level dynamics of gene regulatory networks [6], which is also related to embryological development and to the organism's history. So, a series of biological findings that were difficult to explain by linear causal dynamics (represented by signalling cascades or by genetic pathways) are approached in a new way. As we are going to see in the next subsection, a genetic mutation will likely be related to cancer through the rearrangement it causes in the connections of a whole regulatory network.

### 1.1. Mutations and cell fate in gene regulatory networks

Systems biology and the gene regulatory network approach have long been known to have interesting evolutionary implications for macroevolution [7], modelling the—sometimes unexpectedly—large or negligible effects of genetic mutations on phenotypes. Any somatic evolution framework must, therefore, take such integration into account [8]. For any biological network—e.g. a gene expression network—the existing regulatory interactions between the various genes or molecules determine the *state space*, i.e. the set of states that are available to the network and the possible transitions and trajectories between them. Most state spaces feature one or more *attractors*, i.e. balanced trajectories or equilibrium points in the state space that, once reached by the network, remain stable also in face of perturbations [6].

In a sense, the creative power of genetic mutation and natural selection is limited by the attractor landscape perspective. Many, if not all, networks that drive cell proliferation are intrinsically equipped with growth suppressive properties: they inhibit or eliminate any immediate selective advantage that mutations in these pathways may otherwise give to the cells: the intrinsic suppressive activity for growth within each pathway is controlled by another pathway, adjusting the proliferative potential of cells. In many cases, then, a precancerous cell that acquires a single mutation in an ONG might be trapped within an evolutionary cul-de-sac, because no particular pathway confers a net selective advantage. On the other hand, a genetic mutation can be related to cancer only through the distortion of the state space it causes, turning cancer into a quite stable attractor state. This claim counters a common assumption in the cancer research community, i.e. that once a relevant mutation occurs in a permissive lineage, clonal expansion inevitably follows. Such commonly held assumption is probably incorrect, by both theoretical and empirical means [9]. There still is evidence for positive selection of oncogenic mutations in normal tissues [10], so the truth is somewhere in between and also the notion of an evolutionary cul-de-sac that cannot be amplified by natural selection is partial and simplified.

Also, the sensitivity of a system navigating through its attractor landscape opens the possibility for the non-genetic origin of tumour and metastatic phenotype. The existence of ‘cancer attractors’ would suggest that the development of tumours is a matter of regulation of gene expression and selection of a stable, pre-existing programme, as is the maturation of the cell type and its differentiation during development [11]. The epigenetic character typical of cancer cells, highlighted by many researchers, would be consistent with this hypothesis and based on theories already developed by Waddington at the beginning of the last century [12]. It would, therefore, be appropriate to speak of cancer as a problem of ‘reprogramming’. Indeed, it can be postulated that embryonic attractors remain present in adulthood, although hardly accessible to cells of the organism in this stage of life. We cannot exclude that these can act as tumour attractors in cases of malignancy development. Thus, ontogeny provides oncogenesis with a starting point [6,13], and this is due to the self-organizing nature of the programmes of gene expression.

One of the fundamental properties of the attractor landscape is multistability, i.e. the ability of the system to go back and forth, moving to specific and stable phenotypes in response to a range of non-specific disturbances, including genetic ‘noise’. Coherent changes in cellular phenotype, underlying the neoplastic progression to the metastatic phenotype, may result from dynamic (switch-like) transitions within entire genome-wide gene regulatory networks. A reversible switch is then possible, and becomes plausible in the attractor model, as the neoplastic phenotype can return to the normal one (more on this below, §4).

The malignant phenotype is not a complete cellular reinvention, but rather one of the states potentially existing in the cell. Current research on mesenchyme,^2^ for example, focuses on the role and expression of mesenchyme-specific genes during development and pathological processes, and the locations and capabilities of mesenchymal stem cells. In the attractor landscape perspective, the mesenchyme phenotype should be considered a distinct cellular programme, more consistent than the sum of the effects of individual genes, which separately encode particular characteristics. In fact, the molecules that can induce a mesenchymal phenotype in transformed cells vary. This fact suggests that the malignant transformation itself causes a change in the behaviour of the regulatory networks rather than a change in the mechanism involved. Mutations can change other molecular factors that contribute to the distortion of the state space and to the shift of the system towards a strange attractor (tumour) [6,14].

If pre-existing attractors explain the ease with which random mutations can quickly produce a wide range of embryonic features, Darwinian selection may gain importance in the progression by modulating proliferation and optimizing cellular survival. In this later phase, increase in cell number is a key factor, because a mutation stabilizes and becomes advantageous within a population of individuals, the dynamics of somatic evolution depending crucially on the interaction of rates of mutation and clonal expansion [2].

### 1.2. From gain of function to breakdown of safety mechanisms and exploitation of organism dynamics

One of the main models of cancer, multistage carcinogenesis, is still based on the view that cancer results after a series of somatic mutations that *knockout* the genetic mechanisms suppressing unregulated cell growth and lead intercellular cooperation to collapse [15].

The idea of intra-organism cooperation is well rooted in the contemporary rethink of the distinction between *individual* and *group* in biology [16–18]: a multicellular organism is a complex organization of cells that cooperate with each other to maintain the integration of the whole, and it is a group with a large amount of internal functional organization that is effectively able to suppress the disruptive tendencies coming from the lower level. The maintenance of a multicellular entity is thus allowed by mechanisms that reduce internal competition and conflicts among individual cells. Some examples of these mechanisms are meiosis, sexual reproduction and differentiation of somatic cells. Large, long-lived species seem to have additional genetic mechanisms to suppress cancer and protect intercellular cooperation. Various types of mechanisms are in place, for example, to remove cells that have undergone a process of abnormal cell division. Some of these mechanisms are cell-autonomous, such as those assigned to the control of cell cycle progression, while others are non-cell-autonomous, heavily relying on signals that constrain the cell to remain within the microenvironment that supports it [19]. Together, these tumour suppressor mechanisms are extremely effective, explaining why cancer occurs less than once in a lifetime, on average, despite the trillions of potentially tumorigenic cells, each the bearer of hundreds of genes potentially responsible for cancer and theoretically subject to a significant number of mutations.

It is interesting to see whether cancer can represent a reverse of the policy mechanism evolved to control the selection at levels lower than the organism [17]. Indeed, some models assert that metastasis does not require new mutations, it only requires cancer cells to take control of complex biological programmes, normally involved in the maintenance of cellular and organ-related physiological processes [20]. A metastatic phenotype, more than being a property acquired during the process, would already, in some way, be present in the cells of the primary tumour. This is the case of the mechanisms of the epithelial–mesenchymal transition (EMT), which plays important roles in normal morphogenesis [21]. An EMT is a process that allows a polarized epithelial cell, which normally interacts with basement membrane (BM) via its basal surface, to undergo multiple biochemical changes that enable it to assume a mesenchymal cell phenotype, which includes enhanced migratory capacity, invasiveness, elevated resistance to apoptosis and greatly increased production of extracellular matrix (ECM) components. In tumour cells, these processes would be used in an aberrant way, allowing cells to assume an invasive phenotype.

The past several decades have seen several advances in the integration of evolutionary thinking into studying cancer. The evolutionary lens has created the need for talking about the adaptive evolution of *cancer defences* both between tissues within the individual and between species that have been influenced by natural selection. In fact, an evolutionary imperative for all metazoans seems to be the suppression of mutant cells that would escape their normal limits and move towards independent clonal expansion. Evolution of multicellularity thus necessitated the development of multiple levels of tumour suppression, limiting selective advantage of initial oncogenic mutations [22–24].^3^ This was one of the lines of reasoning leading to the idea that the neoplastic phenomenon should be understood in the framework that evolutionary biologists call ‘multilevel selection’.

## 2. A grand unified view: cancer, the evolution of cooperation and the major evolutionary transitions

As Ellen Clarke wrote, ‘The biological hierarchy did not spring into existence fully formed. It is itself the outcome of a long process of evolution’ [26, p. 315]. Michod already wrote that ‘The evolution of multicellular organisms is the premier example of the integration of lower-level individuals (cells) into a new higher-level individual’ [27, p. 8613]. How did intercellular cooperation come about? What traces, if any, does the organism bear of such evolutionary origin? And is cancer rooted into this deep evolutionary architecture?

Cell biologist Lynn Margulis [28] was a pioneer in proposing that eukaryotic cells evolved by symbiotic associations of bacterial cells (prokaryotes), rather than by accumulation of mutational steps from bacteria [28]. In this view, what we usually call ‘individual’ (namely individual organism) is actually an evolved functionally integrated group of other individuals at a higher level, as in any holobiontic whole [29]. A series of relevant contributions in the philosophy of biology [30–34] generalized Margulis' concept in order to account for other major transitions in evolution (MTE), ranging from groups of functionally organized molecular interactions in early living beings to the evolution of human societies, including the emergence of the first bacterial cells, multicellular organisms and social insects colonies. This approach is now routinely combined with multilevel selection and evoked and used to explain reciprocal coordinations that pervade organisms.

The evolutionary transitions approach confers a fundamental role to cooperation in the deep history of biology. The multilevel selection (MLS) hypothesis has been proposed since the 1980s in order to explain some phenomena such as altruism among animals^4^ of the same species or sterility in social insects. Inclusive fitness is a measure [36,37] that shows how, in determinate conditions, the sharing of particular genotypes can prevail on the survival of the individual: paradoxically, a self-sacrificing behaviour can be the fittest if it protects several related individuals with a high percentage of genotype sharing (*kin selection* [38]).

Both the MTE issue and the MLS theory involve interactions among levels of selection. So, the MLS theory is widely accepted as a theoretical framework for the study of MTE, even though the MTE's perspective considers the balance between levels of selection in a more dynamic way (i.e. balance can itself evolve) than the MLS theory [39,40].

### 2.1. Major evolutionary transitions

The debate on evolutionary transitions has been exposed mainly in Maynard Smith & Szathmáry's *Major Evolutionary Transitions* [31]. The aim of that book is to analyse the six major transitions in evolution: the transition from unlinked replicators to chromosomes; from RNA to DNA; from prokaryotes to eukaryotes; from asexual to sexual replication; from protists to animals, plants and fungi; from individuals to colonies; and from primate societies to human societies. Even if we can discuss the importance of the transitions described by that book, the main idea is that selection favours the grouping of individuals, giving rise to larger entities. It is a process in which a new individual arises from a group of previously existing individuals. In fact, entities cooperate among them to give rise to a new integrated entity, which can better face a new environment. Transitions are, therefore, explained through the selective advantage of replicators.^5^ Cooperation, integration and unitary reproduction are then the characteristics of a successful transition.

An evolutionary transition can, therefore, be defined as a process in which entities, which were capable of independent replication before the transition, are no longer capable of replicating independently after the transition. As a common pattern, during each transition, a series of smaller free-living units, capable of independent replication, give place to a larger unit, thus creating a new level of organization. Cells which form an organism, for example, cannot replicate outside it. This is why there are simple, unaggregated individuals (e.g. bacteria) and more complex individuals (eukaryotic cells), and then very complex biological systems (such as ourselves!), which are composed of trillions of cells. This would also explain why biological individuals are more and more complex throughout evolution.^6^ Increase in size and complexity is what characterizes the major transitions in evolution, in which genetic information is transmitted from one generation to the other.

A core question of evolutionary transitions is *how* a group of individuals might become a new individual, i.e. how does the passage from groups *of* organisms to groups *as* organisms occur, and why lower-level units sacrifice their individuality to be functional parts of a larger body. Granted that the newly formed entity becomes the new unit of selection, or more precisely a unit of fitness [41], does aggregation take place under the pressure of natural selection? Or is the process of emergence of a new level of biological organization non-selective (as in the case of Margulis' symbiogenetic theory),^7^ but resulting in a new potential unit of selection at a higher level?

### 2.2. Multilevel selection

In a multilevel selection perspective, the evolution of multicellular organisms could be seen as the outcome of a competition between selection acting at the lower level (among cells within groups) and selection at a higher level (among multicelled groups). While the former tends to disrupt the integrity of the emerging multicelled organism, the latter may favour the evolution of adaptations for suppressing internal contention. If between-group selection is able to evolve such mechanisms that suppress the potential for disruptive selection, it can become the primary evolutionary force and groups may evolve as novel higher-level organisms by increasing their internal functional organization. Individuals and groups, collectives and particles, become relative terms, both historically and logically, depending on the focal level of analysis. At any level of the biological hierarchy, groups can evolve functional organization to the extent that selection operates at their level and it can develop mechanisms that suppress disruptive forms of selection within groups [18,32,43]. As Michod says, ‘The individuality of multicellular groups is a complex trait. Following Darwin and his approach in *The Origin of Species* to understanding an organ of such complexity as the human eye, we reduce the complexity to a set of evolutionary steps involving simpler traits, each advantageous by itself. In the case of the evolution of multicellular individuals, these stages might involve the formation of cell groups, the increase of cooperation within cell groups, the evolution of conflict mediators to protect the group against cheaters, the increase in group size, the specialization of cells in essential fitness components of the group, and the spatial organization of these specialized cell types’ [27, p. 8613].

The drive to elaborating the multilevel selection theory came from seemingly maladaptive traits. Hamilton focused his attention on unselfish behaviour, a trait that is in apparent opposition to the ‘struggle for existence’ among organisms, i.e. natural selection. According to Hamilton, as altruism exists, then it must have been positively selected for. To explain this trait, which is prima facie unlikely, Hamilton coined the neologism ‘inclusive fitness’, i.e. the fitness one organism expresses in the form of offspring carrying its own genes. Inclusive fitness is a count of the number of ‘offspring equivalents’ an individual rears, rescues or otherwise supports through its behaviour (regardless of who begets them). The individual's own child, who carries one half of the individual's genes, is defined as ‘one offspring equivalent’. Other individuals have different degrees of relatedness, so their safety affects the fitness of the considered individuals. A sibling's child, who will carry one-quarter of the individual's genes, is 1/2 offspring equivalent. Similarly, a cousin's child, who has 1/16 of the individual's genes, is 1/8 offspring equivalent. Crucial to this approach is the attribution of fitness to a single allele rather than to the whole genotype. In Hamilton's model, the evolutionary process ultimately depends on leaving, in a population, as many copies of the individual's genes as possible. The methodology adopted, then, consists of counting the probability each trait has to be reproduced and to survive in the subsequent generations. An altruistic behaviour can have a high probability to appear again in the following generations if an organism—through such behaviour—helps close relatives (likely bearers of the same trait) to survive.^8^

Several observations can be explained as a result of selection inside the group and over the whole group. Among such observations we have—under certain conditions—altruistic traits and mechanisms preserving post-reproductive individuals. Natural selection can favour health at youth or middle age (high reproductive value) over health at old age (low reproductive value). This means that, all else being equal, selection for cancer suppression should dramatically drop after reproductive age. In species with significant parental investment, however, the reproductive value of older individuals or even those past reproductive age may derive from inclusive fitness. A prediction descending from this is a correlation between variation in parental investment levels and variation in cancer susceptibility across species [24,25]. Kin selection and parental investment would create an explanatory selective pressure in this respect, still allowing cancer risk increase with size and longevity within species.

Generalized multilevel selection (MLS) can be further generalized to account for MTE. In the framework proposed by Okasha [40], in the first phase of evolutionary transitions, selection acts on single entities; therefore, fitness is aggregative. This is named multilevel selection 1 (MLS1). After the transition, selection acts on the whole aggregate so that the group will have its own fitness, which will not be entirely dependent on its elements' fitness. This process is named multilevel selection 2 (MLS2). Evolutionary transitions are, thus, an example of a passage from MLS1 to MLS2.

### 2.3. Unification under the somatic evolution view?

Many authors have valued the Evolutionary Somatic view of cancer for its broad unification potential, making sense of many phenomena by considering the healthy organism as a context of selective processes that are antagonistic to the cohesion and functioning of the organism. The supposed cellular Darwinian selfishness of cancerous growth would be an example that such lower-level selection was never entirely eliminated: cancer would be an imbalance between selection processes at different levels in a multilevel selection view. Godfrey-Smith talked about ‘de-Darwinization’ of lower levels to describe the Major Transitions in evolution.^9^ If multicellularity evolved due to a mechanism of de-Darwinization, we might say that cancer cells are re-Darwinized. In fact, somatic selection in this view is also constantly *reducing* the risk of cancer: many potentially harmful mutations also increase the probability of triggering apoptosis, hence initially lead to cells with reduced net proliferation rates. But somatic selection also acts to promote, not only to prevent, cancer.

As we have already seen, cancer can be seen as a breakdown of intercellular cooperation: cells mutate to phenotypes of uncoordinated proliferation. Somatic evolution would exploit the accumulation of mutations in the cells of the body (soma) during its life cycle, which would underlie ageing too [44,45]. All the main phases of cancer would be characterized by somatic selection, i.e. carcinogenesis, tumour development and metastasis. Metastasis would, thus, be the endpoint of a long process of selection, i.e. of the sustained change over time in a population of cells due to heritable differences that make a difference to relative survivorship and reproductive success. The dissemination of tumour cells in the organism would take place through a cascade of subsequent events [46] in a process called ‘colonization’, which ends up with the metastatic invasion of new organs and tissues.

The Evolutionary Somatic model of cancer has indeed a great unification potential, it was a productive assumption for cancer research, and has found some supporting data and especially simulation models. However, it is also contradicted by persistent difficulties and contrasting information that are rarely brought to their ultimate critical epistemological implications: that the ‘early features’ pre-tumoural features of cells, as well as the existence of an ever-ongoing selective process, could be a back-projection of features that are relevant in a particular phase, metastasis, to earlier phases that might follow different rules.

## 3. Problems with the somatic selection view of carcinogenesis

Under the somatic evolution hypothesis of cancer presented above, at the cellular level there is selection for those cells that manifest greater survival and proliferative capacities. Natural selection among cells seems to nicely fit with the multilevel selection view in the major transition framework: the achievement that took place in deep time is still with us, and can be lost in the present time, reverting cells to a state similar to their ancestral state. However, is this really possible? Does the intra-organism context provide the conditions for intercellular selection? For sure, the hypothesis of cancer as an imbalance between selection processes at different levels of organization is something distinct from the major transition approach as they refer to different temporal scales: in principle, ultimate causes and proximate causes could be disjunct; if intercellular cooperation would never have emerged without some kind of multilevel selection, that does not automatically mean that cells in a multicellular organism are easily converted to a Darwinian population.

### 3.1. Cancer stages and crossing microecological conditions

A possible problem with a comprehensive somatic evolution model of carcinogenesis is that the inherent *locality* of natural selection does not seem to be taken into great consideration. Selective pressures can hardly be the same across all phases of cancer. Fitness has to be considered a value that is always relative and extremely sensitive to a given context, composed, for example, of physical and climatic conditions,^10^ amount and structure of available space, cohabiting species which can create phenomena such as mimicry, symbiosis, commensalisms, parasitism or classic competition for food resources. All these aspects are susceptible to continuous and important changes, and what is favoured in particular conditions may not be such elsewhere. To conform to such locality, evolutionary models of cancer articulate evolutionary theory in terms of fitness versus ‘real adaptation’—which would go beyond the local immediate context. The terminological uncoupling of fitness and adaptation, however, does not solve the problem.

While, for example, *tumour* cells compete for resources like oxygen and glucose and for space, it is not clear whether *precancerous* cells do the same. Early events of tumorigenesis are difficult to measure and identify clinically although, assuming a somatic evolution of cancer, they could be simulated in a principled way [47–49].

There is a problem of actually *identifying* supposed ‘metastatic properties’ acquired in a former local cellular microenvironment. The various sub-clones that are formed by the first selective process do have peculiar characteristics such as, for example, drug resistance and the ability to give rise to metastases, eventually getting to a more aggressive neoplastic phenotype. However, during the stochastic evolution of cancer, it has proved difficult to establish a direct causal relationship between environmental and specific genetic/epigenetic factors: for example, chronic inflammation, which can be, in most cases, responsible for initiation and cancer progression, is both microenvironmental alteration and an alteration linked to genetic instability [50].

The constancy and relevance of metastatic properties (e.g. independence from external growth signalling, motility, proliferation, escape from immune recognition, lack of programmed death or senescence) is, however, a minor problem for an Evolutionary Somatic model of cancer. A more fundamental problem lies in the way metastatic properties emerge, i.e. whether they can be considered complex adaptations as opposed to simple one-step activations of pre-existing phenotypic possibilities.

### 3.2. How ‘paradigmatically Darwinian’ are intra-organism cell populations?

Lewontin [51] classically formalized three necessary and sufficient conditions for natural selection: (i) variation, (ii) inheritance and (iii) fitness. According to the evolutionary argument, somatic cells would be able to undergo natural selection, because (i) they are arranged in local populations that exhibit variation, (ii) they transmit their genetic and epigenetic features to daughter cells and (iii) their variations may affect their persistence and proliferation, conferring relative selective advantages [51].

Godfrey-Smith [17] shifted the perspective, pointing out that the crucial element for an evolutionary process by natural selection is a Darwinian *population* [17]. In order for a group of individuals to be a Darwinian population, some criteria have to be fulfilled: variation, heredity and differences in reproductive success. Through these features, we can recognize a Darwinian population, which is a collection of entities linked by a particular parent–offspring relation [52]. According to Godfrey-Smith, however, a population can be more or less ‘paradigmatically Darwinian’, varying along different dimensions and creating more or less suitable conditions for natural selection to appear and be effective. According to Germain [53], intra-organismal cell populations do meet the minimal requirements for natural selection, yet they are not paradigmatic Darwinian populations. For Germain, even if it is uncontroversial that natural selection acts on cells populations, natural selection does not have enough explanatory power. The crucial issue for any explanation resides in the proper consideration of its conditions of validity. Natural selection acts in a population that is ‘paradigmatically Darwinian’, but the existence of such a population cannot be taken for granted in the organism. This context makes sense of some difficulty and arbitrariness in identifying proto-tumoural (and even tumoural) features in the precancerous cells that are supposedly ‘naturally selected’. There is, of course, experimental and epidemiological evidence for positive selection for certain pre-tumoural features,^11^ but most of the studies done in experimental systems do not offer an ideal recapitulation of precancerous tissues and do not look at differential fitness explicitly. Remarkable studies about the initial steps in the somatic evolution of a solid cancer [55,56] focus on very specific contexts in the organisms (such as the bottom of the intestinal crypt, where stem cells are continuously replacing each other in a random fashion) and explore the effect of known mutations (in genes *APC*, *KRAS*, *P53*) on ongoing proliferative dynamics.

Any adaptive evolution is unlikely in a non-paradigmatically Darwinian population. By adaptive evolution, we mean the multi-generational emergence of complex traits as a result of a cumulative selection.^12^ Cancer cells do show complex traits, such as high integration and capacity, to develop blood vessels. Yet, cancer cell ‘adaptations’ usually consist of immediate possibilities offered by their molecular architecture.^13^ For example, melanoma resistance to vemurafenib is due to a loss of exons: this loss of exons cannot be considered a complex adaptation, it is just a little change which does not require any accumulation [53, p. 804]. Signals which are generally used by the healthy organism are co-opted by the tumour mass: for example, in order to grow and produce metastasis, cancer cells use angiogenesis, that is the physiological process through which new blood vessels form from pre-existing ones. Moreover, as we will see (§3.3), these traits are triggered by—and crucially depend on—the microenvironment and tissue organization. For all these reasons, Germain & Laplane [58] consider that tumour level selection does not have an important explanatory role for understanding the somatic evolution of human cancer. Even if cancer progression has some resemblances with MLS2 and even if it seems to act as a major transition, this does not give a big contribution to its understanding as a pathological phenomenon.

We have seen that intra-organism cell ‘adaptations’ can be questioned. Let us now consider the *reproductive* fitness of individual cells. Fitness is a complex concept that translates into different, non-coincident measurements [59,60]. Reproductive fitness concerns the number of offspring, and/or how much the traits of a focus individual will be represented in the next generation. Fitness is always *relative* to a given environment. However, Godfrey-Smith pointed out that in a paradigmatic Darwinian population, the fitness of any individual is *intrinsic*, i.e. it depends more on intrinsic traits than on contingent external factors. How much a cancer cell replicates, instead, depends strongly on extrinsic factors such as position, dimension and nutrients [45]. Cancer cells are normalized relatively to their context, that is they are transformed by their environment according to the tissue they belong to. This feature compromises the possibility of considering cancer cell populations as paradigmatically Darwinian populations, where evolution of complex adaptations by natural selection can take place, and of considering a cancer cell as a paradigmatic Darwinian individual [53].^14^

### 3.3. Context dependence of cell properties

Since the genetic turn in the 1970s, the firm goal of cancer research was the search for key mechanisms and elements (e.g. genes) that could become the target of treatment. In the philosophical field, *specificity* has been largely discussed along with other notions such as stability and level of explanation [62–70]. Close to Woodward's definition [71], which adheres to the most general use made in experimental design, *specificity* is the approximation of a causal relation to the ideal of one-cause-one-effect. *Stability*, instead, has to do with whether a causal relationship continues to hold under changes in background conditions.

In cancer research, *in vitro* cultures remained the privileged experimental system, to some extent favoured by the long-lasting impossibility to study single cells and by the difficulties to deal with the whole organism. *In vitro* cultures were known to be sufficiently homogeneous to assume that all the units they contained were causally efficient and equivalent. Cell lines, established *in vitro*, do not offer a suitable experimental model, as they reduce the complexity of phenomena observed *in vivo*: the equivalence between cell culture results and those obtained in growing animals, which are ultimately a reiteration of the phenomenon to be understood, can often be regarded as incorrect. Yet, the experimental setting favoured a predominance of specificity.

Over the last decades of cancer research, difficulties related to the multiplicity of causal factors have increasingly challenged the specificity attributed to molecular parts in cancer [50]. It became clear that the reconstruction of the functional context of the tissue microenvironment provides a key condition for causal specificity to be studied. Progressively, contextual factors—which include long-range interactions and topological factors—were acknowledged in their role of *stabilizing* the structural and functional properties of molecular parts [13]. The architecture of normal tissue is, indeed, a three-dimensional organizing system that carries positional and historical information. Robustness of networks, reversibility of the effects linked to epigenetic regulation, tissue architecture and genomic analysis gained importance. The modelled organization of normal tissue and the progression of morphogenetic change linked to diffusion phenomena showed how the destruction of morphogenetic gradients is sufficient to provide the aberrant cell phenotype [72]. Established dynamics take over the control of the tumour cells' behaviour: the cell is freed from gradient-based control, irrespective of the presence, or absence, of genetic mutations in cancer cells, during the initial neoplastic process. Both association patterns and cell types change as tissues and organs are formed [73–75].

In the process of scientific explanation, *specificity is secondary to stability* [50]. The stochastic evolution of cancer makes it very hard to establish direct causal relationships with *specific* genetic or epigenetic features. Reconstructing discrete stages is difficult, and attributing the origin of cancer to a unique intracellular molecular component or specific exogenous factor seems unrealistic. This does not mean that we cannot find genetic or epigenetic features that are strongly and ubiquitously related to the risk of developing cancer (e.g. the *BRCA1* mutation, methylation of p16 locus). The point about specificity and stability is deeper: in living beings, strong correlations between genetic features and organizational phenomena, such as cancer, are not to be interpreted as signs of context independence of the involved mechanisms; after all, even the link between a genotype and a phenotype is not a linear function, instead involving a norm of reaction, so context dependence of the consequences of particular genetic/epigenetic changes is to be expected. Moreover, somatic cells in metazoans will arguably have a wide reaction norm, as the genome has to encode a wide range of phenotypic manifestations. From this point of view, cancer can be considered a disease of the ongoing systemic organization of an organism of its natural dynamism. Parts lose their integrated functional properties and become more rigid falling into apparently functional states that require a lower level of energy to be maintained.

A way to capture the pathologic feature of tumour cell behaviours is, thus, the stability/specificity entanglement. Without functional stability, there are no specific functions that allow for explanatory specificity. This ‘stability wins over specificity’ perspective is also interpretive of the numerous studies showing that cancer cells can return to normality when placed in a normal microenvironment and maintain their ability to undergo apparently correct differentiation, despite genetic defects [76–79]. The changes in the genome would then be causally specific only in the context of global destabilization of gene expression.^15^ During the neoplastic process, the molecular components are mainly unvaried, but *their functional activity* changes due to internal and external factors that eventually involve multiple DNA-damaging events. Such change is considered dysfunctional as far as it does not respond to the normal regulative factors properly (e.g. aberrant differentiation) and brings about a change of the subsystems (e.g. genetic instability).

Interpreting cancer as a problem of epithelial–mesenchymal interactions that become pathological by a breakdown of the basic rules that govern tissue organization leads to the idea that tissue-level autonomous mechanisms dominate, and are mediated by, cell–cell interactions rather than by gene–gene relationships. The dichotomy is warranted by ‘causal asymmetry’: while changes in gene interactions affect cell–cell interactions (albeit in a buffered and nonlinear way), the opposite does not hold, as cell–cell communication and gene expression are embedded in a larger context that regulates the properties and behaviour of the cell, without necessarily affecting particular gene sequences, molecular mechanisms or biochemical pathways. Factors that induce a change in the geometric shape of cells are involved in the change of cell fate, influencing the evolution towards apoptosis, quiescence, proliferation or differentiation. As a further factor of complexity, the immune system was shown to play more important roles than identified genetic alterations [83–89].

Many authors have started to conclude from the current state of cancer research that the somatic selection approach seems more adequate to explain surviving events in metastasis, than to enhance the appearance of new stable phenotypes and the transmission of a stable genomic pattern showing a real adaptive behaviour [50].

## 4. Fields for intercellular cooperation

As we have seen, believing that the multistep process that produces a metastatic cancer is the somatic analogue to natural selection encounters more than one difficulty. In addition, due to the irreversible nature of genetic mutation, it is also difficult to explain how certain metastases go back to normal phenotype, especially if inserted in normal surrounding tissues [90,91]. Several different approaches, which are not based on an individual cell, have been proposed to reconcile such behavioural and evolutionary inconsistencies.

In cancer research, we find many examples and experimental evidence that the most important properties of a cancer cell emerge from properties that can be attributed to more inclusive wholes, such as tissues and ‘cells + surroundings’ ensembles. The maintenance of a *status quo* in adult tissues requires that newly generated cells ‘adopt the appropriate fate’ and contribute to the structure and function of the organ to which they belong. Such dynamic stability takes place by ‘dynamic and reciprocal’ exchanges of information between cells and their surroundings [92,93]. Tissues and organs are embedded in an ECM/BM that provides them with structural support and contextual information along with soluble factors. In the same way, tumours exist in intimate relationship with the surrounding microenvironment, and ‘it is the dynamics of this heterogeneous and ever changing ecosystem that provides additional but crucial information for mutated genes to exert their function’ [94, p. 40]. This view of the properties of cancer cells has confirmed the limits of single-cell studies. Take, for example, the property of ‘drug resistance’. Drug-resistant cells were assumed to emerge as winners in the competition after prolonged exposure to cytotoxic agents; they were thought to be the bearers of multiple mutations that fuelled both tumour growth and clinical multidrug resistance. Now it is clear, from new epistemological assumptions and from empirical evidence, that the solid tumour microenvironment/architecture may, in fact, significantly contribute to the emergence of therapeutic resistance [50,94]. Microenvironment and tissue architecture shape selective pressures experienced by tumour cells, much like ecological variables influence selection in natural populations.

An interesting example from cancer biology is the tissue organization field theory (TOFT) that considers some properties of the tissue as more fundamental than some properties of the component cells. For more than 15 years now, cancer researchers Sonnenschein & Soto [95] have been pushing the TOFT of carcinogenesis, according to which neoplasia arises from a problem of three-dimensional organization of a tissue rather than from a normal cell gone awry by mutation or by other mechanisms. For the TOFT, carcinogenesis takes place at the tissue level of biological organization: the appearance of a tumour^16^ is due to chronic abnormal interactions between the mesenchyme/stroma and the parenchyma of a given morphogenic. When the structure of a tissue is affected, cells are ‘disoriented’ and no longer constrained, they cannot differentiate properly and they revert to the default state of all cells which is proliferation (and migration). Conversely, carcinogenesis is a reversible process, whereby normal tissues (or their components) in contact with neoplastic tissues may normalize the neoplasy. The modes in which cells are organized in a tissue are thus causally and explanatorily relevant, so that in the crucial phases of cancer onset, aberrant stimuli affecting the coordination and structure of the hierarchical organization of cellular systems are sufficient and more explanatory than genetic mutations. Tissue-level properties such as fields,^17^ for example, are attributed causal priority over parts and held accountable for carcinogenesis and for tumour heterogeneity.

Soto and Sonnenschein in their work, especially in their book *The Society of Cells* [95], say that the social behaviour of cells, or, on the contrary, their antisocial behaviour as seen in cancer, is not due to the individual genetic set-up of each cell, but is rather due to the ‘field’ in which cells are embedded, which is, in turn, created by the organization of the tissue. According to Soto and Sonnenschein's TOFT, prosocial or antisocial behaviours are not necessarily individual properties, do not necessarily depend on vertical inheritance, but they can emerge as collective ecological properties yet being manifested in the individuals. With TOFT, Soto and Sonnenschein also dissolve the necessity of explaining the abnormal proliferation of a cell: in fact, they assume proliferation as the default state of a cell. In other words, proliferation is automatic, and the cell is oriented by the surrounding field to remain in a disciplinate and quiescent state. Carcinogenesis may be triggered from a shift in the ecological conditions of the tissue, not directly related to any particular genetic mutation, and be reversed.

TOFT and somatic evolution are sometimes contrasted in a dichotomical way. The cell-centred and gene-centred perspective is indeed able to identify real causes, as in the case of the Rb gene and retinoblastoma or the APC. But the relevance of APC or p53 in cancer risk can be explained in the TOFT as a tissue-mediated cause. In hereditary tumours, the role of the context and of the organizational interrelations is implicit in the proper functional definition and identity of the genes. In other words, the TOFT, by keeping a firm focus on the wider context where cancer develops (the tissue and the organism), calls for a conceptual shift from a view of the cancer cell *achieving* a number of functions to a view of the cell that *loses the capability to cooperate* effectively with the other cells in the tissue and in the body. The ‘advantage’ for the single cell would come from a loss—more than an acquisition—of functions.^18^

Therefore, cell-centred and gene-centred perspective can be—to some extent, i.e. as far as the gene-centred perspective works—considered a specific case of the TOFT when it is brought into a unitary epistemological perspective, which is systemic. Sporadic cancers are more appropriately explained by a theory focused on the tissue, like the TOFT. For heritable cancers, a genetic account seems inescapable, but such account finds its conditions of validity in the broader context of the tissue (or the organ or the organism, whichever is more explanatorily relevant).

## 5. Conclusion

The Darwinian paradigm is a unifying theory for several biological phenomena. In cancer research, Vineis *et al*. suggest that the term ‘Darwinian’ needs to be used cautiously, ‘being a short cut for ‘somatic cellular selection’’ [98, p. 1703]: it has entered into use in cancer literature, but ‘it should not be used to imply that Darwinian selection at the population (rather than cellular) level is involved in carcinogenesis’ [98, p. 1704]. As we have seen, evolutionary thinking is widespread in cancer research, but the somatic selection model has serious shortcomings and a domain of validity that is probably limited. Some authors, pointing out the difference between step-wise mutation models and Darwinian evolution, argue that evolution is often explained away rather than taken into consideration [99]. Somatic selection is certainly important in metastasis, and there are cases in which cancer growth and progression seem in line with a somatic selection model [55,56], but something different—related to the organism's dynamic stability—must take place in carcinogenesis and in the early phases. A particular implication of field-based theories and models is that the *explanandum* of carcinogenesis becomes quiescence, and *explanantes* are found at the field level. In any case, the evolution of cooperation—especially the useful concepts of inclusive fitness, kin selection, multilevel selection and major transitions—remains part of that grand theoretical framework that makes sense of all biology: the same theoretical framework which could also explain, on large temporal scales, the evolutionary emergence of multicellular organisms from single-celled ones.

## Footnotes

1 The six functional characteristics attributed to genetic alterations spelled out by Hanahan & Weinberg are as follows: (i) ‘Self-sufficiency in growth signals’: this refers to the observation that tumour cells produce their own growth factors, not depending on external growth signals; (ii) insensitivity to antigrowth signals: normal cells are maintained in a state of quiescence by growth inhibitory signals; genetic changes confer tumour cells the ability to ignore these signals; (iii) evasion of apoptosis: normal cells will activate the self-destruction or apoptosis programme in response to irreversible DNA damage, insufficient growth signals or ONG overexpression, while tumours acquire the means to ‘evade apoptosis’, resulting in an accumulation of altered cells; (iv) ‘Limitless replicative potential’: the majority of mammalian cells generally proliferates for a limited number of times due to the progressive shortening of the chromosome ends or telomeres; virtually all malignant cells have acquired the ability to maintain their telomeres; (v) ‘Sustained angiogenesis’: cancer cells promote the formation of blood vessels; this is essential for the tumour, because cells cannot survive at a distance of more than about 100 µm from blood vessels; (vi) ability to branch out through invasion and metastasis: during the development of most tumours, primary cancer cells acquire the ability to spread from inside the surrounding tissues to distant sites, giving rise to secondary tumours in remote organs. If these genetically determined functional characteristics describe the malignant phenotype, the pathways that cells undertake to arrive at a malignant phenotype, however, are variable, and the order in which the ‘hallmarks of cancer’ manifest themselves can change from tumour to tumour.

2 Mesenchyme is a type of tissue composed of loose cells embedded in the extracellular matrix (a mesh of proteins and fluid), which allows mesenchymal cells to migrate easily and play a crucial role in the origin and development of morphological structures during early development (especially those concerning connective tissues, from bones and cartilage to the lymphatic and circulatory systems). The interactions between mesenchyme and another tissue component, epithelium, help to form nearly all organs in the body.

3 The presence of cancer suppression mechanisms in metazoans is related to the so-called Peto's paradox. The paradox highlights the contrast between a prediction of the multistage model and what is observed in nature [25]. The prediction is that cancer risk should increase with body size and longevity. But this is not observed across species. So why don't large animals have a higher lifetime incidence of cancer than small animals?

4 As Dawkins vividly described, ‘The commonest and most conspicuous acts of animal altruism are done by parents, especially mothers, towards their children. They may incubate them, either in nests or in their own bodies, feed them at enormous cost to themselves, and take great risks in protecting them from predators. To take just one particular example, many ground-nesting birds perform a so-called “distraction display” when a predator such as a fox approaches. The parent bird limps away from the nest, holding out one wing as though it were broken. The predator, sensing easy prey, is lured away from the nest containing the chicks. Finally, the parent bird gives up its pretense and leaps into the air just in time to escape the fox's jaws. It has probably saved the life of its nestlings, but at some risk to itself’ [35, p. 6].

5 ‘The transitions must be explained in terms of immediate selective advantage to individual replicators’ [30, p. 8].

6 Note that the authors’ idea that evolution gives rise to complexity is quite controversial: in some environments, it could be more evolutionarily advantageous to be simple than to be complex. Complexity should, thus, not be seen as a necessary outcome of evolution.

7 Margulis' conception stressed the importance of cooperation in evolutionary processes, in contrast to conflict usually underlined in the Darwinian perspective of selection [42].

8 A more radical ‘gene's eye’ view was later proposed by Dawkins [35]. Each trait is related to a particular gene. Consequently, fitness can be studied in regard to a particular gene, expressed as phenotype traits. In this view, gene-based traits appear as the main actors of the evolutionary process: evolution is described since its beginning as a competition among replicators, i.e. what we currently call genes. These replicators build organisms (vehicles) with the only aim of generating as many copies of themselves as possible and are expressed in the phenotype as all kinds of traits (including unselfish and altruistic ones).

9 ‘Their independent evolutionary activities are curtailed, constrained, or suppressed by what is happening at the higher level—a partial “de-Darwinizing” of the lower-level entities’ [17, p. 122].

10 A classical textbook example is the melanism of the peppered moth *Biston betularia*.

11 For example, UV exposure and ionizing irradiation have been unequivocally linked with expansion of clones with *TP53* mutation—one of the most common and most understood of oncogenic mutations in turn related to reduced capability to undergo programmed cell death is favoured in the context of certain environmental insults [54].

12 Lewens [57] expressed well the concept in his documented conceptual analysis of adaptation and adaptationism: ‘What is distinctive in Darwin's explanation of the facts of apparent design is not simply the idea that fitter variants will replace the less fit ones in a population. It is the idea that through the accumulation of many small, favourable mutations, the emergence of complex adaptations becomes far more likely than it would be through single saltation’ [57, p. 167].

13 Mutational and heritable epimutational changes either unlock some phenotypic features that are not accessible to normal somatic cells of a given tissue (such as those related to embryonic development) or create novel wiring, which is not encountered in normal cells (such as mutations in *RAS*, or many of the fusion oncogenes that create a kinase with abnormal activity that leads to phosphorylation levels not encountered in any normal context). That is why, in Germain's words, ‘Evolution by natural selection plays a role in neoplastic progression and in the acquisition of resistance, but ‘the fundamental problem of cancer’, if there is such a thing, is more a problem of cells whose inner workings have vulnerabilities’ [53, p. 807].

14 According to Lean & Plutynski [61], however, we could say that understanding cancer cells as Darwinian individuals depends on the scale of analysis. Multicellular organisms are hierarchically organized, and every dysfunction, such as cancer, has to be understood in relation to a specific context. Therefore, it might be correct to describe cancer as a dysfunction at a cellular level *and* as a shift of some mechanisms from organism to cellular level.

15 On this point of particular interest is the work done by Capp [80]. Special attention has been also devoted to the demonstration that genetic instability itself (therefore, the accumulation of mutations) follows the onset of an abnormal microenvironment, as studies seem to demonstrate the genetic instability of stem cells, when grown without control of the microenvironment [81]. The same could happen in pre-malignant cells, after the loss of the stabilizing effects from the organization of surrounding tissue. The subsequent deregulation of the DNA maintenance pathways, generated by alteration of the microenvironment, would be sufficient to generate the defects observed in cancer cells, so mutations that inactivate specific genes involved in cell differentiation may be, more generally, a consequence of the other non-mutational mechanisms. While here I focus on the context-dependence of the *effects* of mutations (i.e. specificity) once they had occurred, the *origin* of mutations can also be considered context-dependent, as epitomized in the remark that, ‘It may be more correct to say that cancers beget mutations than it is to say that mutations beget cancers’ [82].

16 Initially and fundamentally, the proponents of the TOFT aim to explain the origin of *sporadic* cancers, i.e. those cancers that seem unrelated to specific inherited genetic mutations. Yet, the TOFT is also proposed as a unifying theory for sporadic and hereditary cancers, since TOFT authors argue that inherited genetic lesions can be explanatorily relevant *as far as* they are related to tissue organization.

17 Actually, Soto & Sonnenschein, citing Gilbert, define a morphogenic field as ‘the *collection of cells* by whose interactions a particular organ or structure forms in the embryo’ ([96], fn. 2, my emphasis). They, thus, seem to be locating a system more than defining a tissue property. Rubin [97] goes more in the direction of defining field in terms of tissue-level properties such as ‘increased saturation density’. Still, fields are ‘grossly invisible, broad regions’. This geometrical/geographical definition meets therapeutical aims, since identifying fields is eventually aimed at excising them along with the tumour in surgery, thereby reducing the possibility of recurrence at the site. The elaboration of the concept of field is one of the many examples of the TOFT calling for a more intense and specific philosophical work to better define its theses and to spell out its deeper implications [50].

18 The definition of function in philosophy of biology is not so easy: a function is usually considered as the result of natural selection. The function of an organ, for example, is the reason why that organ has been selected [87]. Anyway, this definition is quite controversial [88]. In any case, the two views of cancer are not alternative, because carcinogenesis is such a complex process that it is difficult to describe it purely in terms of achieving or losing functions.

## Data accessibility

This article has no additional data.
